# Patient Preferences for Using Remote Care Technology in Heart Failure: Discrete Choice Experiment

**DOI:** 10.2196/68022

**Published:** 2025-11-05

**Authors:** Ahmed Al-Naher, Jennifer Downing, Dyfrig Hughes, Munir Pirmohamed

**Affiliations:** 1 Institute of Systems, Molecular and Integrative Biology (ISMIB), University of Liverpool, Liverpool, United Kingdom; 2 Institute of Population Health, University of Liverpool, Block B: Waterhouse Buildings, 1-5 Brownlow Street, Liverpool, L69 3GF, United Kingdom, 44 (0) 151 795 5422; 3 Centre for Health Economics and Medicines Evaluation, Bangor University, Bangor, United Kingdom

**Keywords:** heart failure, telehealth, remote care, engagement, discrete choice, medical devices

## Abstract

**Background:**

Remote care technology has been used to bridge the gap between health care in a clinical setting and in the community, all the more essential post-COVID. Patients with chronic conditions may benefit from interventions that could provide more continuous and frequent monitoring of their disease process and support self-management. A common barrier, however, is the lack of engagement with technological interventions or devices that provide care remotely, which could lead to loss of resources invested and reduced quality of care.

**Objective:**

This discrete choice experiment elicits the preferences of patients with heart failure with regard to potential remote care technologies that they would be willing to engage with and, in turn, creates a hierarchy of factors that can affect engagement for use within future technology design.

**Methods:**

A survey was created using discrete choice design and with input from a patient and public involvement group. It was distributed online via social media to patients with heart failure and to patient support groups. The attributes used for the experiment were based on a previous systematic review looking at factors that affect engagement in remote care and which generated five central themes, each of which was assigned to an attribute directly: communication (increasing interaction between patients and health care staff/carers/other patients), clinical care (improving the quality of care compared to established practice), education (providing tailored information to help with self-care and reduce uncertainty), ease of use (the technical aspects of the intervention are easy to handle without issues), and convenience (the intervention fits well around the patient’s lifestyle and requires minimal effort). Each of the five themes had two levels, positive and negative. The survey presented participants with multiple forced-choice two-alternative scenarios of remote care, which allowed them to trade attributes according to their preference. The results were analyzed using binary logit to obtain preference weights for each attribute.

**Results:**

A total of 93 completed responses were entered into the analysis. The results of the binary logit created coefficients for each attribute, which equated to the relative preference of the associated themes: clinical care, 2.022; education, 1.252; convenience, 1.245; ease of use, 1.155; communication, 1.040. All calculated coefficients were statistically significant (*P*<.01).

**Conclusions:**

The results show that, in this cohort of patients with heart failure, the most preferred factor, clinical care, has enough value to be traded for approximately any two other factors. It also shows that the factor of communication is the least preferred attribute. Technology designers can use the associated preference weights to determine the relative increase of value perceived by patients by adding in certain attributes, with the greatest gains achieved by prioritizing clinical care. This would result in increased engagement in a chronic heart failure population that would benefit most from remote care.

## Introduction

Heart failure is a chronic and progressive condition, which is defined as the inability of the heart to pump sufficient blood to meet the body’s oxygen demand. This is often caused by structural cardiac conditions that reduce the efficiency of the heart, for example, ischemic heart disease, because it weakens cardiac muscle and reduces the pump’s effectiveness. Other associated conditions can contribute to the disease severity, such as diabetes mellitus and hypertension, which promote structural changes to the heart, or chronic obstructive pulmonary disease, which reduces the blood’s oxygen-carrying capacity. The result is a complex clinical syndrome that causes symptoms of fatigue, shortness of breath, and peripheral edema. As this usually occurs in an older patient cohort with an average age of 76 years with multiple comorbidities, their clinical management is complex and their health care needs are high. They typically have reduced mobility, cognition, and mood and face challenges in self-care and efficacy [1].

Remote care technologies can gather clinical data remotely, which enables closer monitoring of patients who are at a high risk of day-to-day clinical variation. These technologies provide easier access to care and have the potential to empower patients to improve self-management, enabling early identification and resolution of severe health issues before they require hospital admission. However, the drop-off rate for these devices is extremely high in this older population. Lack of engagement with the device may result in failure to achieve the anticipated improvements in clinical outcomes and could lead to a significant waste of time as well as research and development costs. This not only burdens patients and their health care providers but ultimately hinders the landscape of technology adoption in chronic diseases, limiting their potential to enhance patient care [2,3]. Therefore, when designing new remote care interventions, it is essential to consider user engagement as the driving force for the uptake and continued use of a remote care device for disease management.

A systematic review of the perceived benefits and drawbacks of remote care, from a clinician, patient, and carer viewpoint [4], identified five common themes that can be used to describe the experiences of users when engaging with remote care technology: communication (increasing interaction between patients and health care staff/carers/other patients), clinical care (improving the quality of care compared to established practice), education (providing tailored information to help with self-care and reduce uncertainty), ease of use (the technical aspects of the intervention are easy to handle without issues), and convenience (the intervention fits well around the patient’s lifestyle and requires minimal effort). While this research concluded that each of the five themes was instrumental to maintaining patient engagement, it did not provide any insight as to which themes were prioritized most by patients. Therefore, to facilitate application of this work in real-world technology design, it is important to quantify the relative hierarchy of the themes and identify which factors could lead to greater engagement in a heart failure cohort.

Choice-based surveys can be used to understand the stated preference of a population for health provision [5]. Here we employ a discrete choice experiment (DCE) approach. In DCE, variables of interest or attributes are traded against one another in different scenarios to ascertain their relative importance [6]. These trade-offs provide information about patient decision-making processes in terms of what attributes participants are willing to compromise on in favor of others, thus understanding their ranked preference. DCEs can be used to simulate uptake or adoption of a new intervention or device based on its characteristics. This can also inform how changes in these attributes can affect user decisions under different scenarios and different values or levels of each attribute to determine to what extent changes should be made for optimal uptake.

We designed a DCE questionnaire to gather primary opinions from patients living with heart failure to elicit their preferences for remote care as categorized by our five themes. The themes capture user experience with minimal overlap and so are amenable to being delineated in questionnaire form, which lends itself well to a choice-based survey [7]. Using each theme as an attribute in the DCE design enables us to quantify the relative importance of each theme to patients with heart failure.

## Methods

### Overview

Since our themes were generated from grounded theory, their titles may be interpreted in a variety of ways. We therefore created clear descriptions for each attribute in relation to remote care (see Table 1). For each attribute, we chose two levels, positive and negative, corresponding to the level of attainment of any given attribute, with neutral included as a negative level [8,9].

**Table 1.** Description of the main attributes and levels used to determine the questions (trials) in the discrete choice questionnaire.^a^

Attribute	Description	Level coding	Level description
Communication	The ability of the technology to create increased contact and follow up between patients and others, including health care staff, family, carers, or other patients	0	Reduces or does not improve opportunities for contact and communication
		1	The technology increases opportunities for contacts and communication
Clinical care	The technology in some way affects the current clinical care given to the patient for their heart failure condition.	0	The technology makes no impact on current clinical care
		1	Improves clinical care from current practice or provides more options for medical management, including providing information to make better decisions on care
Education	The impact of the technology on patients’ knowledge about their health and self-care	0	There is no improvement in knowledge or ability to self-care
		1	The technology provides details that clarify and provide useful information to the patient about their condition and aid in their self-care and management
Ease of use	The intuitiveness and relative ease that the technology can be introduced and used by new users, including technical difficulties and jargon	0	The technology is overly complex, with little technical support and may have a high rate of technical difficulties and complications, or is difficult to access for new users
		1	The technology is easy and intuitive to use, requires relatively little support, or is easy to understand and use by a wide audience
Convenience	The measure of how much time and effort is saved by the use of the technology compared to normal care. Also relates to the level of comfort afforded by the technology in the patient’s home.	0	There is no difference in the amount of time and effort required for self-care actions, or the device creates more work for the patient and requires extra time to use, or it creates increased worry or stress
		1	The device functions to save time, such as automating processes or providing relevant information at the right time, and results in less work for self-care actions or allows the patient to be more comfortable in their own home environment

a Attributes were taken from themes generated from a systematic thematic analysis of factors affecting user engagement with remote care technology in a population of patients with heart failure [4].

### Questionnaire Construction

Each question forced the participant to choose between two hypothetical remote care technologies with opposing attribute levels, that is, a positive level in an attribute in one choice means that the alternative choice will have a negative level of that same attribute. The forced choice design reduced the complexity of adding an opt-out alternative to each question, which minimized questionnaire fatigue [10].

The choice sets (the combination of levels of each attribute that were grouped together per question) were assigned based on a predetermined, orthogonal design algorithm [11]. For a discrete choice questionnaire containing 5 attributes each with 2 levels, this resulted in 32 profiles split across 16 questions. The order of the questions was randomized to mask the pattern of the choice sets. The attributes were listed in alphabetical order in each question [12,13].

### Sample Size

We used an established method for determining the minimum sample size for conjoint analyses [14]:


$$N>\frac{500\times c}{t\times a}$$

Where *N* is the minimum sample size; *c* is the number of levels; *t* is the number of questions; and *a* is the number of alternative answers.

For a 16-question survey with 2 choices, the recommended minimum response size is 32 participants. We took this as a minimum and left the online survey open until the end of the study window to capture as many responses as possible.

### Criteria for Patient Participation

Patients who were aged 18 years or over and had a diagnosis of chronic heart failure were included in the study. Exclusion criteria included (a) diagnosis of acute heart failure without any chronic component and (b) non-English speaking patients (the questionnaire was only available in English).

### Patient and Public Involvement

A patient participation group consisting of five patients with heart failure and related conditions was formed to aid the outputs of the research. These patients were recruited via a free access public engagement event held at the University of Liverpool on October 23, 2017. This event involved talks from cardiology and technology experts to inform on upcoming heart failure technology research and generate interest in public participation. After the formation of the group, several informal discussions and feedback sessions were conducted between February and March 2018, where the group piloted the questionnaire and had input into the patient information leaflet. Design changes were made due to this feedback, including shortening the questions, formatting for better readability, as well as expanding on the patient information leaflet to provide more context for the study (Multimedia Appendix 1 and Multimedia Appendix 2). Furthermore, the patient group members helped to suggest places where the survey could be distributed online to heart failure care communities.

### Ethical Considerations

As per HRA guidance [15], responses to online surveys imply consent as long as participants are provided with sufficient information to reach an informed decision. We worked with our patient group to develop substantially descriptive participant information for them to make an informed choice. This study was approved by the Research Ethics Committee at the University of Liverpool (ref: 3314). The survey was exported online using a secure digital platform (SurveyMonkey), which complies with EU Privacy Laws and General Data Protection Regulations, and is registered under the Data Protection Act. This online platform was used to create a web link, which was the primary means of distributing the survey to participants. In accordance with the principles of data minimization and purpose limitation under General Data Protection Regulations, no personal or demographic data were collected by the research team; therefore, participants were not identifiable, nor was there any direct contact between the research staff and participants. No monetary compensation was offered to any participant for completing the questionnaire. Raw and processed data were stored securely on encrypted university intranet servers.

### Survey Distribution

The link to the survey was distributed to national and international heart failure patient groups, which were accessed via social media and communications through heart failure charities. A list of organizations approached for distribution can be found in the supplementary information (Multimedia Appendix 3). It is important to note that while the study was conducted in the United Kingdom, the survey was distributed worldwide, and so the respondents were not limited by geographic location.

### Analysis

Responses were analyzed using limited dependent-variable models to determine preference weights of each attribute [16]. From this, we can infer which attributes participants are willing to trade in favor of others. Our DCE is a forced-choice, five-attribute, two-level, two-alternative questionnaire (Multimedia Appendix 4). As both the choices and the levels were binary, binary logit [16] was used to determine the likelihood of the outcome. The logarithmic function ensures the likelihood values are constrained between 0 and 1 [17].

The logit definition is as follows: [18]:

Logit(*P*)=log(odds)=log(*P*/(1−*P*))

As part of the regression, we assign logit(P) as a linear function of any given attribute *Xi*, so that:


$$\log\frac{P}{\left(1-P\right)}=\alpha +\beta Xi=Ui$$

Where *P*=probability (of choosing this option); *α*=reference value or constant; *β*=coefficient of attribute *X*; *i*=attribute number; *U*=utility

The logit value is proportional to the odds of an attribute, affecting the probability of choosing an alternative. Thus, these values can be compared directly as preference weights for each variable. The preference value for each attribute is known as utility, which is the measure of importance of each attribute or combination of attributes. In order to standardize for participant heterogeneity, random effects were added to create a mixed binary logit model [18,19].

The utility value of each combination of attribute level was obtained by adding the constant coefficient of attribute *X* from the logit model, with the coefficients of each positive attribute present. The odds were obtained by exponentiating the utility. To convert this to percentage uptake probability, that is, the likelihood of choosing this remote care device as opposed to the alternative, we divided the Odds by 1+Odds [20]. The dataset was analyzed using RStudio version 1.0.136. These calculations were also corroborated using STATA/MP 13.0.

## Results

The survey was open for 133 days (June 3, 2018–October 14, 2018) and was initiated by 164 participants. The completion rate was 57%, giving 94 completed responses. A limited trial of the paper questionnaire was undertaken in local heart failure clinics, but this generated only 1 completed response. To verify accuracy and consistency of the extracted results, visual inspection was undertaken to assess for discrepancies and anomalous data, and all survey attempts with missing or incomplete responses were excluded. Response nondifferentiation was identified, and two responses were omitted due to nontrading (all responses from a participant were either choice A or choice B). This left 93 valid responses. (Multimedia Appendix 5)

We identified some positive attribute dominance in the responses (respondent always chose the option with a positive level in a single attribute) [21]: 10 participants had positive dominance for clinical care, three for education, two for ease of use, and one for communication. There were no cases of negative attribute dominance. The main outputs of the mixed binary logit are displayed in Table 2.

**Table 2.** Results from the binary logit analysis of the discrete choice questionnaire^a^.

Attribute	Coefficient (95% CI)	*P* value
*Intercept*	−3.357 (−3.654 to −3.060)	<.001
Clinical care	2.022 (1.810 to 2.233)	<.001
Education	1.252 (1.077 to 1.428)	<.001
Convenience	1.245 (1.053 to 1.436)	<.001
Ease of use	1.155 (0.982 to 1.327)	<.001
Communication	1.040 (0.864 to 1.216)	<.001

a The coefficients for each attribute represent relative patient preference weighting for that attribute in isolation, relative to the intercept. Higher value coefficients represent a proportional increase in preference by patients with heart failure.

Each coefficient was highly statistically significant, indicating that there was a sufficient sample size and significant effect of each attribute on patient choice. The goodness of fit was evaluated using the pseudo R-squared of the logit model, which showed a value of 0.1833. The attributes presented in the model thus explain 18% of the variance in choice of each participant, a typical result for a DCE of this size [22].

We calculated the utility value, odds ratio, and percentage probability of choosing each combination of attribute levels (Table 3). The utility represents the preference value for choosing each alternative and can be compared for evaluating complete choice sets (different combinations of attributes). This contrasts with coefficient values for each attribute, calculated from the logit model, which indicates preferences for individual attributes (Textbox 1).

**Table 3.** A comparison of all 32 possible combinations of attributes and levels that can be applied to a remote care intervention.

Communication	Clinical care	Education	Ease of use	Convenience	Utility	Odds	% uptake probability
1	1	1	1	1	3.36	28.70	96.63
0	1	1	1	1	2.32	10.14	91.02
1	1	1	0	1	2.20	9.05	90.05
1	1	1	1	0	2.11	8.27	89.21
1	1	0	1	1	2.10	8.20	89.13
1	0	1	1	1	1.34	3.80	79.17
0	1	1	0	1	1.16	3.20	76.17
0	1	1	1	0	1.07	2.92	74.49
0	1	0	1	1	1.06	2.90	74.35
1	1	1	0	0	0.96	2.61	72.26
1	1	0	0	1	0.95	2.59	72.11
1	1	0	1	0	0.86	2.36	70.26
0	0	1	1	1	0.29	1.34	57.32
1	0	1	0	1	0.18	1.20	54.50
1	0	1	1	0	0.09	1.09	52.26
1	0	0	1	1	0.08	1.09	52.07
0	1	1	0	0	−0.08	0.92	47.93
0	1	0	0	1	−0.09	0.91	47.74
0	1	0	1	0	−0.18	0.83	45.50
1	1	0	0	0	−0.29	0.74	42.68
0	0	1	0	1	−0.86	0.42	29.74
0	0	1	1	0	−0.95	0.39	27.89
0	0	0	1	1	−0.96	0.38	27.74
1	0	1	0	0	−1.06	0.35	25.65
1	0	0	0	1	−1.07	0.34	25.51
1	0	0	1	0	−1.16	0.31	23.83
0	1	0	0	0	−1.34	0.26	20.83
0	0	1	0	0	−2.10	0.12	10.87
0	0	0	0	1	−2.11	0.12	10.79
0	0	0	1	0	−2.20	0.11	9.95
1	0	0	0	0	−2.32	0.10	8.98
0	0	0	0	0	−3.36	0.03	3.37

a The table compares each intervention's relative utility, odds ratio and percentage uptake probability values, which can each be considered as composite preference weights of the combination of all attribute levels in a remote care intervention.

Textbox 1. **How to use the data for comparative analysis as a worked example.**

The percentage uptake probabilities are derived from the calculated utility score and so are symmetrical, giving a probability of 50% to an intervention with a utility score of 0. As such, they are not intended to be used in isolation but mainly as a means of calculating marginal differences in engagement between two comparator intervention states.

To compare engagement between two different types of intervention, for example, with and without a certain attribute included, we can use Table 3 to calculate the marginal probability, which is the difference in percentage uptake probability between the two interventions. This can be done by choosing the two rows that most correspond to each individual remote care device (based on present attributes) and then subtracting the percentage uptake probabilities from each other to get the marginal probability.

For example, in a remote care intervention with no attributes present (row: 0/0/0/0/0), the percentage uptake probability is 3.37%. An intervention that has the attribute of communication alone (row: 1/0/0/0/0) has the percentage uptake probability of 8.98%. Therefore, the marginal probability gained by adding the communication attribute to the intervention which has no attributes is calculated as 8.98−3.37=+5.61%.

Alternately, to work out the marginal probability of adding clinical care instead, we look to the row which only includes the clinical care attribute (row: 0/1/0/0/0) to see that its percentage uptake probability is 20.83%. We then subtract this from the percentage uptake probability of the intervention with no attributes: 20.83–3.37=+17.46%.

The marginal probability figure can be regarded as the change in utility between comparator interventions and represents the amount of value added in terms of engagement by altering the remote care intervention to meet specific additional attributes. At a glance, it can therefore be seen that the value added from incorporating the clinical care attribute is much greater than adding the communication attribute to an intervention without either.

Taking the mean of marginal probabilities for adding the attribute to each permutation which excludes it gives another quantitative measure of patient preference. We found the mean marginal probabilities per attribute to be as follows: communication=+18.04%, ease of use=+20.1%, convenience=+21.8%, education=+21.9%, and clinical care=+37.6%. These values could also be interpreted as the average relative increase in patient preference gained by adding this attribute to an intervention that lacks it. This is a useful measure for comparing the value of the attributes themselves against each other; however, for a more detailed comparison of combinations of attributes (whole interventions), the marginal probability described in the above calculation would be more suitable. For example, mean marginal probabilities suggest patients would be more likely to value adding clinical care outcomes to an intervention (+37.6%) compared to adding communication to an intervention (+18.0%) on average. However, if the aim is to compare an intervention with no attributes and one which has both clinical care and communication, the specific marginal probability between these interventions can be calculated more precisely. Refer to the row that contains both clinical care and communication (row: 1/1/0/0/0) to see that the percentage uptake probability for this intervention is 42.68%. Then calculate the difference between this and the percentage uptake probability of the intervention with no attributes as in the examples above (row: 0/0/0/0/0). This gives a marginal probability of 42.68−3.37=+39.31%. Thus, the specific marginal probabilities are ideal to be used when there is a fixed intervention state, or a starting point, such as a design or existing intervention that is intended to be improved upon.

## Discussion

### Principal Findings

The analysis ranked the remote care attributes in the following order of importance for patients with heart failure: (1) clinical care; (2) education; (3) convenience; (4) ease of use; and (5) communication. Based on the coefficients of the logit fit, clinical care was almost twice as important as the lowest scoring variable, communication. Remote care technology design should therefore prioritize clinical care improvements first and foremost. The attributes of education and convenience had similar preference values, which were around 20% greater than communication. Ease of use was 11% more important than communication. This pattern of preference shows a disproportionately high preponderance toward clinical care, with the second, third, and fourth ranked attributes plateauing at a similar level. Therefore, if a trade-off is required, any other attribute may be sacrificed for the sake of preserving clinical care, while still incentivizing patient engagement.

### Comparison to Prior Work

A number of DCE and conjoint analyses have been published regarding patient preferences for telecare since the COVID pandemic [23-25]. However, there have been no other DCEs evaluating engagement of remote care technologies in this patient cohort of chronic heart failure. Therefore, the study provides a valuable insight into the factors of remote care devices that encourage engagement. In a post-COVID era, remote care technologies have gained greater importance in health care. Patients with heart failure are a vulnerable cohort and so are more likely to be offered remote consultation. Therefore, these preference rankings are all the more vital at this time to help remote care become better established in medical practice for those that need it most.

### Strengths and Limitations

#### Methodological Design Advantages

Among the advantages of our experiment was that each possible combination of levels and attributes was presented to the participants, resulting in a full factorial design. This establishes a more accurate statistical value for each preference as fewer assumptions are made. By contrast, partial factorial designs sacrifice comprehensiveness for brevity [26].

Another strength is that the attributes used were based on evidence from a grounded theory qualitative systematic review, specific to the subject [4]. This means that the outputs of the review were tailored to this questionnaire design, resulting in relevant attributes derived from high-quality evidence.

#### Questionnaire Considerations

Our study does have some limitations. First, the statistical model assumes each participant will always choose the option that maximizes their utility, which could lead to bias. We tried to mitigate this by adding a random effect to model heterogeneity of preference choices, even if they might be irrational (or of less utility). This study, therefore, presents the preference values in terms of a probability of choosing each option, which means the likelihood of a nonrational choice still exists.

Second, the DCE assumes that the participant is equally attentive on question 1 as they are on question 16, and this may not always be the case. The complexity of the questions coupled with their repetitive nature may contribute to participant fatigue when answering questions. We had the option of creating either an 8-question design or a 16-question design. We opted for the latter to obtain a greater statistical effect from each respondent. In hindsight, this may have contributed to the high non-completer rates [27].

Third, in many DCEs, the alternative choices are based on existing interventions or ones that are ready to market. In this study, we asked participants to imagine theoretical technologies. This enables the outputs to be applied to a wide variety of technology designs in the future. A disadvantage is the potential for hypothetical bias, which can lead to a discrepancy between patient stated preference and the actual (or revealed) preference [28].

Fourth, related to this hypothetical scenario is the fact that an opt-out option was not presented to participants. This forced-choice design meant that they were not able to express dissatisfaction with both alternatives at once. We recognize that this is an artificial scenario, and in reality, participants may be disinclined to engage with either option. However, the aim of this study was to understand the ranking preferences of patient behavior rather than whether they would engage in any specific intervention. Thus, the design of the study was adapted to maximize the depth of information, at the cost of using hypothetical scenarios.

Finally, there was a lack of a third *neutral* level for each attribute: either the attribute was present in the remote care technology or it was not. This means that there was no *neither* option for the participant to choose to indicate that a specific attribute was unimportant in their decision-making. Furthermore, the negative level was often used to effectively indicate two different levels by specifying both an *absence* and *negative* effect of the attribute within the meaning. Although we chose to omit the neutral level from the questionnaire design, the benefit of this is that it allows the analysis to be more straightforward in terms of the binary logit analysis rather than adopting a multinomial logit model, which requires more assumptions [29]. Another benefit to the two-level system was that the choice burden on the participants was minimized which likely improved completion rates.

#### Generalizability

First, the effects of the recorded attributes are presented in relation to one another, which means that the assessments of value lack generalizability outside of the context of the comparison versus each other in a heart failure cohort. It is important, therefore, to realize that these results may not translate to cohorts with other conditions, or even other chronic diseases, and that the results do not have intrinsic value independent from the attributes they are compared to here. A mitigating factor is that the analysis relies on the foundation of its supporting research to substantiate the list of tested attributes. The supporting research is a thorough and in-depth look at lived experiences within this cohort and seeks to be as comprehensive as possible while capturing commonalities in themes that can be of value in the assessment of technology in this chronic condition [6].

Second, the online self-selection method may reduce the generalizability of the study findings to other cohorts such as in-person heart failure clinics. It was likely also completed by those with greater digital access and skills. However, in a post-COVID era, where patients are more likely to be familiar with remote care, those lacking digital access and skills may be in the minority. Our findings should nevertheless be interpreted within the context of patients who are generally supportive of new technologies [30].

Third, the methodology used in this study resulted in a lack of demographic data collection. This may also deter from the generalizability of findings. While the heart failure demographic is generally well established, the self-selection and timing of the questionnaire, as well as its online distribution route, have the potential to skew the responses based on whether the participant sample was seen to diverge from the average heart failure demographic, for example, to those younger, with less comorbidities, living in more affluent locations. Without the demographic data to put these results into context, the generalizability of the outputs when applied to a new cohort of patients with heart failure may be affected. However, since the attributes were built from a rigorous analysis of patient experience data generated from a variety of patient demographics and geographical locations, we posit that the central themes continue to have relevance across a wide range of patient populations.

Finally, the factor of *cost*, which is normally assessed in this manner by means of adding an attribute that asks how much the participant is willing to pay for certain factors, is missing. The remote care intervention that participants were asked to envision was hypothetical, and therefore there is no real-world cost to incorporate in the assessment. The same can be said for other real-world factors such as management, administration, and access to the intervention. This may potentially lead to inaccurate responses as the hypothetical scenarios may pose unrealistic cost choices with reduced credibility effect, leading to invalid willingness to pay estimates [31]. However, it is worthwhile considering that cost implications and access restrictions played a role in defining the attribute of *ease of use* in the original thematic synthesis, as high cost and maintenance requirements of the device contributed to poor accessibility of the intervention and was seen to impact the ease of use for patients [32].

### Future Work

Improving this and similar surveys may require shifts in methodologies to make it more generalizable, albeit with additional feedback. In the first instance, while patient participation was a key determinant in the design of the methodology, additional value may have been obtained by reaching out to technology designers and start-ups that create devices within the space. Obtaining this kind of feedback would enable tailoring of the outputs in such a way as to provide the most deliverable benefit in the context of future design by, for instance, presenting realistic alternatives grounded in existing technologies as opposed to theoretical ones.

To mitigate some of the limitations further, it may be also useful to obtain demographic information and location of participants so as to correctly contextualize the responses based on patient profile, recognizing that different subpopulations may have differences in preference.

Finally, in order to address noncompletion rates, the questionnaire could be shortened in order to be less mentally taxing, while also ensuring a process of gathering feedback from participants as to reasons for noncompletion.

### Conclusions

Our questionnaire used a DCE method to elicit preferences for remote care technology from patients with heart failure from around the world. Results of the analysis indicate that clinical care was substantially more valued as a factor for engagement with remote technology than the four other themes of education, convenience, ease of use, and communication. Our findings also allow approximations of increase in engagement by sequentially adding in these individual factors to an existing remote care device based on their preference values. This hierarchy could provide useful insights for technology designers to check the effectiveness of an intervention’s features in engaging the end user and help develop a plan of improvement for devices based on their missing attributes. Incorporating these attributes appropriately will ultimately bring remote care technology to these patients in a more effective and engaging manner, to reduce the burden of morbidity from chronic heart failure.

## Supplementary material

10.2196/68022 Multimedia Appendix 1 Participant information sheet for discrete choice experiment.

10.2196/68022 Multimedia Appendix 2 Instructions for answering questionnaire.

10.2196/68022 Multimedia Appendix 3 Charities, organizations, and social groups contacted for online questionnaire distribution.

10.2196/68022 Multimedia Appendix 4 Discrete choice experiment questionnaire.

10.2196/68022 Multimedia Appendix 5 Online questionnaire responses.

## Abbreviations

DCE: Discrete choice experiment
MRC: Medical Research Council
NIHR: National Institute for Health Research
