# Supplementary material for: Patient Preferences for Using Remote Care Technology in Heart Failure: Discrete Choice Experiment
Source: JMIR Cardio. 2025 Nov 5;9:e68022. doi: 10.2196/68022 (PMC12588585; doi:10.2196/68022)
Supplement: Multimedia Appendix 1 [file cardio-v9-e68022-s001.docx]

**Participant information sheet for discrete choice experiment**


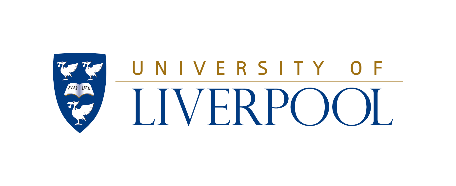


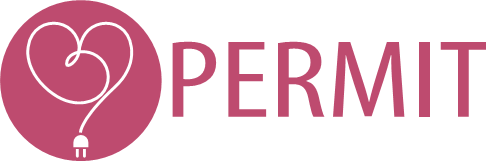


Questionnaire on remote care in heart failure

**Participant Information**

Thank you for your interest in heart failure research.

This questionnaire is designed to find out which parts of remote care technology are most important for patients with heart failure.

**Am I** **eligible?**

To be eligible to complete this survey, you need to be over 18 and have been diagnosed with any form of long-term heart failure, including cardiomyopathy, infective or congenital causes.

**What is Remote Care?**

Remote care is the use of technology to help carry out medical care that would normally be available in a hospital or a clinic, but within your own home instead. Examples include a wireless blood pressure reader or a digital scale that sends readings directly to the doctor from your home. As medical technology continues to improve, remote care is becoming more common in medicine as a way to look after long-term health conditions from home. It helps care for patients that have trouble travelling to appointments, and can also alert doctors early if a patient needs more care, so that they can be treated quicker.

**Why Heart Failure?**

We have chosen patients with heart failure because this is a serious condition which often affects patients over a long period of time. Patients with heart failure often have other health conditions too, that make their care more complex so these patients would greatly benefit from improved health monitoring at home.

**How will this survey** **help patients like me?**

With the knowledge from this study, we can begin to design new technologies that suit heart failure patients better, so that they can continue to be used for longer and provide the greatest benefit for patients such as yourself. Remote care will soon have a much bigger role in medical care, and this research is an important way of bringing it closer to patients by finding out what your preferences are. The results of this study will directly help us create life-saving devices for heart failure, which could be used by you in the near future.

**How do I complete** **the questionnaire?**

The questionnaire will consist of 16 questions which should take no longer than 15 minutes to complete. You will be presented with an example of a remote care technology, and will be given two options that describe various features of the technology. For each question, you will be asked to pick which of the two technologies (A or B) you would prefer to use, based on the descriptions provided.

**What will happen to my data?**

All data is anonymised and confidential. This survey will not ask any personal questions or any information that could be used to identify you. The data will be stored on the online server for as long as the survey is open (3 months from the start date of the survey). The data will then be downloaded to a secure university account, which is only accessible by the researchers. As your information is fully anonymised, we unfortunately cannot contact you with the results of the study. However the study will be published on completion and you can visit our website below to find out any new project updates.

**Who is conducting** **the study?**

This study is supported by the University of Liverpool, UK and is funded by the Collaboration for Leadership in Applied Health Research and Care North West Coast (CLAHRC NWC) which is a part of the National Institute of Health Research (NIHR). This project is part of the Personalised Renal Monitoring via Information Technology (PERMIT) study. Find out more about the project here: <http://tinyurl.com/NIHR-PERMIT>

The main contact is Dr. Ahmed Al-Naher at the University of Liverpool. For further questions and enquiries, please contact: [aalnaher@liverpool.ac.uk](mailto:aalnaher@liverpool.ac.uk).

**What is the** **PERMIT project?**

Personalised Renal Monitoring via Information Technology (PERMIT) is a research project aimed to reduce the effects of kidney disease in heart failure. Unfortunately, patients with heart failure have a higher chance of developing problems with their kidney later on in life, and since this has no symptoms in the early stage, it is not often detected until it causes permanent kidney damage. PERMIT aims to create better guidelines for monitoring kidney health, to detect disease early and prevent damage that could lead to kidney failure. Developing remote care technology is a good way of detecting this damage at an early stage, and this study will help us achieve that.
