# Supplementary material for: Patient Preferences for Using Remote Care Technology in Heart Failure: Discrete Choice Experiment
Source: JMIR Cardio. 2025 Nov 5;9:e68022. doi: 10.2196/68022 (PMC12588585; doi:10.2196/68022)
Supplement: Multimedia Appendix 2 [file cardio-v9-e68022-s002.docx]

**Instructions for answering questionnaire**

**How to answer this questionnaire**

Imagine that you have been provided with a home monitoring system by your doctor to keep track of your health and manage your heart failure. A home monitoring system includes all of the following devices:


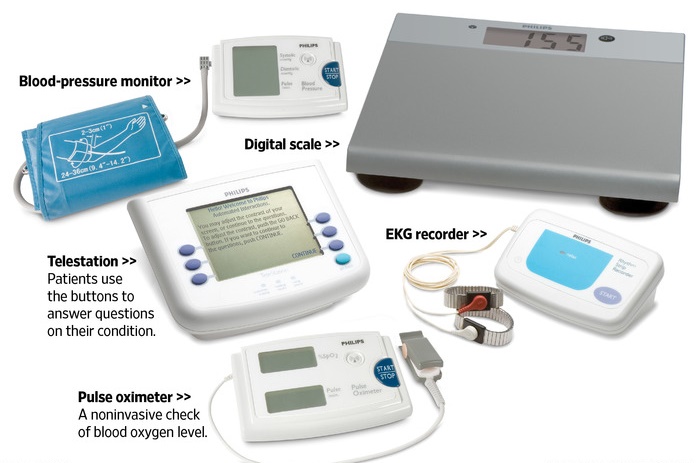


This system of devices is designed to allow you to take your own blood pressure, weight, oxygen level and heart rhythm at home, and track your symptoms. The readings are displayed on a website that you can use to send information back to your doctor to monitor your health.

This questionnaire is designed to find out which features of the home monitoring system are most important to you. Each of the following questions will describe two different versions of this home monitoring system. Please choose one of the two home monitoring systems you would most prefer to use.

Each question will include two options to choose from. Each option consists of 5 descriptions about the home monitoring system. This information will re-appear in each question, but will be different between the A and B choices, and in a different order each question. This is done on purpose to identify a pattern in your preferences, and see which technologies are more important to you than others.

As an example, in question 1, you will be presented with the two options in this way:

Example Question 1.

Which out of monitoring system A or B would you prefer?

|  | Monitoring system A | Monitoring system B |
| --- | --- | --- |
| How often are measurements checked by your doctor, and how does this influence your care? | Frequent:  used by your doctor to guide treatment | Infrequent:  has no impact on the treatment you receive |
| Does the home monitoring system have a way to talk with or receive information directly from your doctor? | No | Yes |
| How long does it take each day to use, and send results to your doctor? | Long time | Quickly |
| How easy is the home monitoring system to use? | Very | Not at all |
| What feedback is given about your measurements, or information about your condition? | None | Some |
| Tick one: |  |  |

Please read the statements of both Monitoring System A and B carefully before deciding between which of the two you would prefer to use. Then select either A or B by ticking the appropriate box. There will be a total of 16 of these questions in total.

While it might feel like these questions are repetitive, they have been very carefully designed using scientific methods to get the most amount of information from your answers, which will in turn be very important for designing new technologies for heart failure patients. The questions become easier to answer the more you complete, and the questionnaire should take less than 10 mins in total. It is important that you complete all of these questions for your response to be counted. Your choices should reflect your personal preference only and so there are no wrong answers.
