# Supplementary material for: Patient Preferences for Using Remote Care Technology in Heart Failure: Discrete Choice Experiment
Source: JMIR Cardio. 2025 Nov 5;9:e68022. doi: 10.2196/68022 (PMC12588585; doi:10.2196/68022)
Supplement: Multimedia Appendix 3 [file cardio-v9-e68022-s003.docx]

**Charities, organisations and social groups contacted for online questionnaire distribution**

Charities and organisations:

- Pumping Marvellous
- Cardiomyopathy UK
- Heart UK
- British Cardiovascular Society
- British Society of Heart Failure
- Heart failure society of America
- British Heart Foundation
- Age UK
- Age concern
- Liverpool Heart and Chest Trust Members Matters

Social media groups (via facebook):

- Heart Failure Aware
- Heart Failure Matters!
- Chronic Heart Failure
- Congestive heart failure support
- Congestive heart failure support group
- Daily strength heart failure support group
- Help medical research chronic heart failure group
- Pacemaker support group
- Young pacemaker patients and supporters
- Pacemaker and ICD support group
- Living with ICD support group
- The hypertrophic cardiomyopathy association.
