# Supplementary material for: Patient Preferences for Using Remote Care Technology in Heart Failure: Discrete Choice Experiment
Source: JMIR Cardio. 2025 Nov 5;9:e68022. doi: 10.2196/68022 (PMC12588585; doi:10.2196/68022)
Supplement: Multimedia Appendix 4 [file cardio-v9-e68022-s004.docx]

**Discrete choice experiment questionnaire**

Question 1.

Which out of monitoring system A or B would you prefer?

|  | Monitoring system A | Monitoring system B |
| --- | --- | --- |
| How often are measurements checked by your doctor, and how does this influence your care? | Frequent:  used by your doctor to guide treatment | Infrequent:  has no impact on the treatment you receive |
| Does the home monitoring system have a way to talk with or receive information directly from your doctor? | No | Yes |
| How long does it take each day to use, and send results to your doctor? | Long time | Quickly |
| How easy is the home monitoring system to use? | Very | Not at all |
| What feedback is given about your measurements, or information about your condition? | None | Some |
| Tick one: |  |  |

Question 2.

Which out of monitoring system A or B would you prefer?

|  | Monitoring system A | Monitoring system B |
| --- | --- | --- |
| How often are measurements checked by your doctor, and how does this influence your care? | Frequent:  used by your doctor to guide treatment | Infrequent:  has no impact on the treatment you receive |
| Does the home monitoring system have a way to talk with or receive information directly from your doctor? | No | Yes |
| How long does it take each day to use, and send results to your doctor? | Quickly | Long time |
| How easy is the home monitoring system to use? | Not at all | Very |
| What feedback is given about your measurements, or information about your condition? | None | Some |
| Tick one: |  |  |

Question 3.

Which out of monitoring system A or B would you prefer?

|  | Monitoring system A | Monitoring system B |
| --- | --- | --- |
| How often are measurements checked by your doctor, and how does this influence your care? | Infrequent:  has no impact on the treatment you receive | Frequent:  used by your doctor to guide treatment |
| Does the home monitoring system have a way to talk with or receive information directly from your doctor? | No | Yes |
| How long does it take each day to use, and send results to your doctor? | Quickly | Long time |
| How easy is the home monitoring system to use? | Very | Not at all |
| What feedback is given about your measurements, or information about your condition? | None | Some |
| Tick one: |  |  |

Question 4.

Which out of monitoring system A or B would you prefer?

|  | Monitoring system A | Monitoring system B |
| --- | --- | --- |
| How often are measurements checked by your doctor, and how does this influence your care? | Infrequent:  has no impact on the treatment you receive | Frequent:  used by your doctor to guide treatment |
| Does the home monitoring system have a way to talk with or receive information directly from your doctor? | Yes | No |
| How long does it take each day to use, and send results to your doctor? | Quickly | Long time |
| How easy is the home monitoring system to use? | Not at all | Very |
| What feedback is given about your measurements, or information about your condition? | None | Some |
| Tick one: |  |  |

Question 5.

Which out of monitoring system A or B would you prefer?

|  | Monitoring system A | Monitoring system B |
| --- | --- | --- |
| How often are measurements checked by your doctor, and how does this influence your care? | Frequent:  used by your doctor to guide treatment | Infrequent:  has no impact on the treatment you receive |
| Does the home monitoring system have a way to talk with or receive information directly from your doctor? | Yes | No |
| How long does it take each day to use, and send results to your doctor? | Quickly | Long time |
| How easy is the home monitoring system to use? | Not at all | Very |
| What feedback is given about your measurements, or information about your condition? | Some | None |
| Tick one: |  |  |

Question 6.

Which out of monitoring system A or B would you prefer?

|  | Monitoring system A | Monitoring system B |
| --- | --- | --- |
| How often are measurements checked by your doctor, and how does this influence your care? | Infrequent:  has no impact on the treatment you receive | Frequent:  used by your doctor to guide treatment |
| Does the home monitoring system have a way to talk with or receive information directly from your doctor? | No | Yes |
| How long does it take each day to use, and send results to your doctor? | Long time | Quickly |
| How easy is the home monitoring system to use? | Very | Not at all |
| What feedback is given about your measurements, or information about your condition? | Some | None |
| Tick one: |  |  |

Question 7.

Which out of monitoring system A or B would you prefer?

|  | Monitoring system A | Monitoring system B |
| --- | --- | --- |
| How often are measurements checked by your doctor, and how does this influence your care? | Frequent:  used by your doctor to guide treatment | Infrequent:  has no impact on the treatment you receive |
| Does the home monitoring system have a way to talk with or receive information directly from your doctor? | Yes | No |
| How long does it take each day to use, and send results to your doctor? | Long time | Quickly |
| How easy is the home monitoring system to use? | Very | Not at all |
| What feedback is given about your measurements, or information about your condition? | None | Some |
| Tick one: |  |  |

Question 8.

Which out of monitoring system A or B would you prefer?

|  | Monitoring system A | Monitoring system B |
| --- | --- | --- |
| How often are measurements checked by your doctor, and how does this influence your care? | Infrequent:  has no impact on the treatment you receive | Frequent:  used by your doctor to guide treatment |
| Does the home monitoring system have a way to talk with or receive information directly from your doctor? | Yes | No |
| How long does it take each day to use, and send results to your doctor? | Quickly | Long time |
| How easy is the home monitoring system to use? | Very | Not at all |
| What feedback is given about your measurements, or information about your condition? | Some | None |
| Tick one: |  |  |

Question 9.

Which out of monitoring system A or B would you prefer?

|  | Monitoring system A | Monitoring system B |
| --- | --- | --- |
| How often are measurements checked by your doctor, and how does this influence your care? | Frequent:  used by your doctor to guide treatment | Infrequent:  has no impact on the treatment you receive |
| Does the home monitoring system have a way to talk with or receive information directly from your doctor? | No | Yes |
| How long does it take each day to use, and send results to your doctor? | Long time | Quickly |
| How easy is the home monitoring system to use? | Very | Not at all |
| What feedback is given about your measurements, or information about your condition? | None | Some |
| Tick one: |  |  |

Question 10.

Which out of monitoring system A or B would you prefer?

|  | Monitoring system A | Monitoring system B |
| --- | --- | --- |
| How often are measurements checked by your doctor, and how does this influence your care? | Frequent:  used by your doctor to guide treatment | Infrequent:  has no impact on the treatment you receive |
| Does the home monitoring system have a way to talk with or receive information directly from your doctor? | Yes | No |
| How long does it take each day to use, and send results to your doctor? | Long time | Quickly |
| How easy is the home monitoring system to use? | Very | Not at all |
| What feedback is given about your measurements, or information about your condition? | Some | None |
| Tick one: |  |  |

Question 11.

Which out of monitoring system A or B would you prefer?

|  | Monitoring system A | Monitoring system B |
| --- | --- | --- |
| How often are measurements checked by your doctor, and how does this influence your care? | Infrequent:  has no impact on the treatment you receive | Frequent:  used by your doctor to guide treatment |
| Does the home monitoring system have a way to talk with or receive information directly from your doctor? | Yes | No |
| How long does it take each day to use, and send results to your doctor? | Long time | Quickly |
| How easy is the home monitoring system to use? | Not at all | Very |
| What feedback is given about your measurements, or information about your condition? | Some | None |
| Tick one: |  |  |

Question 12.

Which out of monitoring system A or B would you prefer?

|  | Monitoring system A | Monitoring system B |
| --- | --- | --- |
| How often are measurements checked by your doctor, and how does this influence your care? | Frequent:  used by your doctor to guide treatment | Infrequent:  has no impact on the treatment you receive |
| Does the home monitoring system have a way to talk with or receive information directly from your doctor? | No | Yes |
| How long does it take each day to use, and send results to your doctor? | Long time | Quickly |
| How easy is the home monitoring system to use? | Not at all | Very |
| What feedback is given about your measurements, or information about your condition? | Some | None |
| Tick one: |  |  |

Question 13.

Which out of monitoring system A or B would you prefer?

|  | Monitoring system A | Monitoring system B |
| --- | --- | --- |
| How often are measurements checked by your doctor, and how does this influence your care? | Frequent:  used by your doctor to guide treatment | Infrequent:  has no impact on the treatment you receive |
| Does the home monitoring system have a way to talk with or receive information directly from your doctor? | No | Yes |
| How long does it take each day to use, and send results to your doctor? | Long time | Quickly |
| How easy is the home monitoring system to use? | Not at all | Very |
| What feedback is given about your measurements, or information about your condition? | None | Some |
| Tick one: |  |  |

Question 14.

Which out of monitoring system A or B would you prefer?

|  | Monitoring system A | Monitoring system B |
| --- | --- | --- |
| How often are measurements checked by your doctor, and how does this influence your care? | Frequent:  used by your doctor to guide treatment | Infrequent:  has no impact on the treatment you receive |
| Does the home monitoring system have a way to talk with or receive information directly from your doctor? | No | Yes |
| How long does it take each day to use, and send results to your doctor? | Quickly | Long time |
| How easy is the home monitoring system to use? | Very | Not at all |
| What feedback is given about your measurements, or information about your condition? | Some | None |
| Tick one: |  |  |

Question 15.

Which out of monitoring system A or B would you prefer?

|  | Monitoring system A | Monitoring system B |
| --- | --- | --- |
| How often are measurements checked by your doctor, and how does this influence your care? | Frequent:  used by your doctor to guide treatment | Infrequent:  has no impact on the treatment you receive |
| Does the home monitoring system have a way to talk with or receive information directly from your doctor? | No | Yes |
| How long does it take each day to use, and send results to your doctor? | Long time | Quickly |
| How easy is the home monitoring system to use? | Not at all | Very |
| What feedback is given about your measurements, or information about your condition? | Some | None |
| Tick one: |  |  |

Question 16.

Which out of monitoring system A or B would you prefer?

|  | Monitoring system A | Monitoring system B |
| --- | --- | --- |
| How often are measurements checked by your doctor, and how does this influence your care? | Frequent:  used by your doctor to guide treatment | Infrequent:  has no impact on the treatment you receive |
| Does the home monitoring system have a way to talk with or receive information directly from your doctor? | Yes | No |
| How long does it take each day to use, and send results to your doctor? | Long time | Quickly |
| How easy is the home monitoring system to use? | Not at all | Very |
| What feedback is given about your measurements, or information about your condition? | None | Some |
| Tick one: |  |  |

Thank you for participating in this survey!
