# Supplementary material for: Patient Preferences for Using Remote Care Technology in Heart Failure: Discrete Choice Experiment
Source: JMIR Cardio. 2025 Nov 5;9:e68022. doi: 10.2196/68022 (PMC12588585; doi:10.2196/68022)
Supplement: Multimedia Appendix 5 [file cardio-v9-e68022-s005.docx]

**Online questionnaire responses**

| Respondent ID | Start Date | End Date | Q1 | Q2 | Q3 | Q4 | Q5 | Q6 | Q7 | Q8 | Q9 | Q10 | Q11 | Q12 | Q13 | Q14 | Q15 | Q16 |
| --- | --- | --- | --- | --- | --- | --- | --- | --- | --- | --- | --- | --- | --- | --- | --- | --- | --- | --- |
| 10278297688 | 2018-10-14 12:01:26 | 2018-10-14 12:13:37 | B | B | A | B | A | B | B | A | B | B | B | B | B | A | B | B |
| 10257831138 | 2018-10-04 15:34:18 | 2018-10-04 15:39:56 | B | B | A | A | A | A | B | A | A | A | B | B | B | A | A | B |
| 10257349524 | 2018-10-04 11:54:05 | 2018-10-04 12:10:15 | A | B | A | B | A | B | A | A | B | A | B | B | B | A | B | B |
| 10257240489 | 2018-10-04 10:45:21 | 2018-10-04 10:56:54 | B | B | A | B | B | A | B | A | B | A | B | B | B | A | B | B |
| 10256132127 | 2018-10-03 21:18:54 | 2018-10-03 21:24:47 | B | B | B | A | A | B | B | A | A | B | B | B | B | A | B | B |
| 10255967147 | 2018-10-03 20:10:47 | 2018-10-03 20:18:46 | B | B | B | B | B | B | B | B | B | B | B | B | B | B | B | B |
| 10255437081 | 2018-10-03 16:55:42 | 2018-10-03 17:09:51 | B | B | A | B | B | A | B | A | A | B | B | B | B | A | B | B |
| 10254348840 | 2018-10-03 7:33:48 | 2018-10-03 7:40:44 | B | B | B | B | A | A | B | A | B | A | A | A | B | A | A | B |
| 10251806692 | 2018-10-02 8:07:25 | 2018-10-02 8:22:18 | B | B | B | B | A | B | B | A | B | A | A | A | B | A | A | B |
| 10249501647 | 2018-10-01 11:50:57 | 2018-10-01 12:06:53 | A | A | B | B | A | B | A | A | A | A | B | A | A | A | A | A |
| 10243823214 | 2018-09-27 18:09:48 | 2018-09-27 18:14:45 | A | A | B | B | A | B | A | B | A | A | B | A | A | A | A | A |
| 10243097507 | 2018-09-27 13:37:35 | 2018-09-27 13:44:03 | A | B | A | B | B | A | A | A | A | A | B | B | B | A | B | B |
| 10242243249 | 2018-09-27 3:53:47 | 2018-09-27 4:43:22 | A | A | B | A | A | B | A | A | B | A | A | B | B | A | B | B |
| 10241360881 | 2018-09-26 19:46:17 | 2018-09-26 20:06:13 | B | B | B | A | A | B | B | A | B | A | A | B | B | A | B | A |
| 10240696157 | 2018-09-26 15:46:36 | 2018-09-26 16:04:30 | A | A | B | B | A | B | A | B | A | A | B | A | A | A | A | A |
| 10240379366 | 2018-09-26 13:53:28 | 2018-09-26 14:00:45 | A | A | B | A | A | B | A | B | A | A | B | A | A | A | A | A |
| 10240366861 | 2018-09-26 13:48:20 | 2018-09-26 13:57:17 | B | B | B | B | A | B | A | A | B | A | A | B | B | B | B | A |
| 10235987662 | 2018-09-24 19:39:45 | 2018-09-24 19:48:14 | B | B | A | A | A | B | B | A | B | B | B | B | B | A | B | B |
| 10235925457 | 2018-09-24 19:12:51 | 2018-09-24 19:25:53 | A | A | B | B | A | B | A | B | A | A | B | A | A | A | A | A |
| 10235392875 | 2018-09-24 15:40:48 | 2018-09-24 15:42:33 | A | A | A | A | A | A | A | A | A | A | A | A | A | A | A | A |
| 10222943592 | 2018-09-18 4:27:40 | 2018-09-18 4:40:42 | A | A | B | B | A | B | A | A | A | A | B | B | B | A | B | B |
| 10219973536 | 2018-09-16 12:16:17 | 2018-09-16 12:23:01 | A | A | B | B | A | B | A | A | B | A | B | A | B | A | B | B |
| 10218747251 | 2018-09-15 3:24:27 | 2018-09-15 3:32:06 | A | A | A | B | A | B | A | A | B | A | B | B | B | A | B | B |
| 10218552357 | 2018-09-14 23:43:35 | 2018-09-15 0:16:24 | B | B | B | B | A | A | A | A | A | A | A | A | B | A | A | B |
| 10215732567 | 2018-09-13 18:32:23 | 2018-09-13 18:45:21 | B | B | B | B | A | A | B | A | B | A | A | A | B | A | A | B |
| 10215409350 | 2018-09-13 16:20:06 | 2018-09-13 16:29:13 | B | B | B | B | A | B | A | A | B | A | A | A | B | A | A | B |
| 10214553556 | 2018-09-13 9:18:05 | 2018-09-13 9:28:51 | A | A | B | B | A | B | A | B | A | A | B | A | A | A | A | A |
| 10214454353 | 2018-09-13 7:58:46 | 2018-09-13 8:15:30 | B | B | B | B | A | A | B | A | B | A | B | B | B | A | B | B |
| 10213989921 | 2018-09-13 1:28:09 | 2018-09-13 1:36:23 | A | A | A | B | A | B | A | B | A | A | B | A | A | A | A | A |
| 10213981474 | 2018-09-13 1:19:28 | 2018-09-13 1:31:41 | A | A | B | B | A | B | A | B | A | A | A | A | B | A | A | A |
| 10213906975 | 2018-09-13 0:26:48 | 2018-09-13 0:41:06 | A | A | B | B | A | B | A | B | A | A | B | A | A | A | A | A |
| 10213769995 | 2018-09-12 23:12:23 | 2018-09-12 23:20:06 | A | A | B | B | A | B | A | B | A | A | B | A | A | A | A | A |
| 10213731360 | 2018-09-12 22:50:43 | 2018-09-12 22:59:21 | A | A | B | B | A | B | A | A | B | A | B | A | B | A | B | B |
| 10213621275 | 2018-09-12 21:53:49 | 2018-09-12 21:58:22 | A | A | B | B | A | B | A | B | A | A | B | A | A | A | A | A |
| 10183927246 | 2018-08-28 15:26:43 | 2018-08-28 15:33:32 | B | A | B | B | A | B | A | A | B | A | B | A | A | A | A | A |
| 10183898446 | 2018-08-28 15:14:41 | 2018-08-28 15:19:15 | B | B | A | A | A | B | A | A | B | B | B | B | B | A | B | B |
| 10180087180 | 2018-08-26 11:08:21 | 2018-08-26 11:34:28 | B | B | B | B | A | B | A | A | A | A | B | B | B | A | B | B |
| 10175274218 | 2018-08-23 5:30:23 | 2018-08-23 5:37:10 | B | A | B | B | A | B | A | B | A | A | B | A | A | A | A | A |
| 10173533086 | 2018-08-22 12:25:32 | 2018-08-22 12:32:39 | B | B | B | B | A | B | A | A | B | A | A | A | B | A | A | B |
| 10173234695 | 2018-08-22 7:35:10 | 2018-08-22 7:55:55 | B | B | B | B | A | B | A | A | B | A | B | A | B | A | B | B |
| 10173204156 | 2018-08-22 7:10:35 | 2018-08-22 7:19:15 | B | B | B | B | A | B | A | A | A | A | A | A | A | A | A | A |
| 10172466377 | 2018-08-21 20:50:24 | 2018-08-21 21:01:26 | B | B | B | A | A | B | B | A | B | A | A | A | B | A | A | A |
| 10172448184 | 2018-08-21 20:40:16 | 2018-08-21 20:52:37 | A | A | A | B | A | B | A | A | A | A | B | B | A | A | A | A |
| 10172371053 | 2018-08-21 20:00:21 | 2018-08-21 20:04:52 | A | A | B | B | A | B | A | B | A | A | B | A | A | A | A | A |
| 10172347427 | 2018-08-21 19:46:03 | 2018-08-21 19:56:25 | B | B | B | B | A | A | A | A | B | A | B | B | B | A | B | B |
| 10171920304 | 2018-08-21 16:17:08 | 2018-08-21 16:26:39 | A | A | B | B | A | B | B | A | B | A | B | B | B | A | B | B |
| 10171897494 | 2018-08-21 16:07:02 | 2018-08-21 16:10:39 | B | B | A | B | A | B | A | B | A | A | B | B | B | B | A | B |
| 10171776934 | 2018-08-21 15:10:53 | 2018-08-21 15:16:52 | B | B | B | B | A | B | A | A | B | A | A | B | B | A | B | B |
| 10171705534 | 2018-08-21 14:36:33 | 2018-08-21 14:46:33 | A | A | A | B | A | A | A | A | A | A | B | B | B | A | B | B |
| 10171700676 | 2018-08-21 14:34:16 | 2018-08-21 14:42:40 | B | B | A | A | A | A | B | A | B | A | A | B | B | A | B | B |
| 10171696364 | 2018-08-21 14:33:06 | 2018-08-21 14:37:15 | B | B | A | A | A | A | B | A | B | B | B | B | B | A | B | B |
| 10171681911 | 2018-08-21 14:26:10 | 2018-08-21 14:30:08 | B | B | B | B | A | A | A | A | A | A | B | B | B | A | B | B |
| 10168745428 | 2018-08-19 20:11:50 | 2018-08-19 20:24:16 | A | B | B | B | A | B | A | A | A | A | B | B | B | A | B | B |
| 10167545781 | 2018-08-18 8:28:55 | 2018-08-18 8:48:37 | A | A | B | B | A | B | A | A | A | A | B | A | A | A | A | A |
| 10167319388 | 2018-08-18 2:02:50 | 2018-08-18 2:10:40 | B | B | B | A | A | B | A | A | B | B | B | B | B | A | B | B |
| 10166900276 | 2018-08-17 20:02:09 | 2018-08-17 20:08:45 | B | A | A | A | A | B | B | A | B | B | B | B | B | A | B | B |
| 10165746667 | 2018-08-17 6:26:43 | 2018-08-17 6:36:50 | A | B | A | A | A | B | B | A | B | A | A | B | B | B | B | B |
| 10164075031 | 2018-08-16 11:48:11 | 2018-08-16 11:55:28 | A | A | B | A | A | B | A | B | A | A | B | A | A | A | A | A |
| 10162439260 | 2018-08-15 15:39:39 | 2018-08-15 15:59:15 | B | A | B | B | A | B | A | B | B | A | B | B | B | A | A | B |
| 10121604877 | 2018-07-20 10:46:02 | 2018-07-20 10:56:58 | A | A | B | B | A | B | A | B | A | A | B | A | A | A | A | A |
| 10114931259 | 2018-07-17 11:26:53 | 2018-07-17 11:33:22 | B | B | B | A | A | B | A | A | A | A | A | A | A | A | A | A |
| 10114853424 | 2018-07-17 10:12:44 | 2018-07-17 10:18:56 | A | A | B | B | A | B | A | A | A | A | B | B | B | A | B | B |
| 10108156950 | 2018-07-12 13:41:24 | 2018-07-12 13:51:31 | B | B | B | B | A | A | B | A | B | A | B | B | B | A | B | B |
| 10108059060 | 2018-07-12 12:25:54 | 2018-07-12 12:37:21 | A | A | B | B | A | B | A | A | B | A | B | B | B | A | B | B |
| 10108046274 | 2018-07-12 12:13:49 | 2018-07-12 12:25:15 | A | B | B | B | A | B | A | A | B | A | A | B | B | A | B | A |
| 10106102137 | 2018-07-11 11:10:00 | 2018-07-11 11:16:19 | B | B | B | A | A | B | A | A | B | A | A | B | B | A | B | A |
| 10104336552 | 2018-07-10 13:08:52 | 2018-07-10 13:27:29 | B | B | B | B | A | B | B | A | B | A | B | A | B | A | B | B |
| 10104122608 | 2018-07-10 9:52:05 | 2018-07-10 10:08:44 | B | B | A | A | A | B | A | A | B | A | A | B | B | A | B | B |
| 10104096699 | 2018-07-10 9:29:11 | 2018-07-10 9:36:55 | B | A | A | A | A | B | B | A | B | B | B | B | B | A | B | B |
| 10103035134 | 2018-07-09 18:11:49 | 2018-07-09 18:21:02 | B | B | B | B | A | A | A | B | B | A | A | A | A | A | A | A |
| 10103014388 | 2018-07-09 17:58:44 | 2018-07-09 18:07:43 | A | B | B | B | A | B | A | A | A | A | B | A | B | B | B | A |
| 10102967909 | 2018-07-09 17:33:28 | 2018-07-09 17:39:03 | B | B | B | B | A | A | B | A | B | A | A | A | B | A | A | B |
| 10102890680 | 2018-07-09 16:53:18 | 2018-07-09 16:57:14 | B | B | B | A | B | B | A | B | B | A | B | A | B | A | B | B |
| 10096680713 | 2018-07-04 12:53:36 | 2018-07-04 13:11:14 | B | B | B | B | A | B | A | B | A | A | B | A | A | A | A | A |
| 10091854549 | 2018-06-30 22:54:05 | 2018-06-30 23:07:10 | B | B | A | B | A | B | A | A | B | A | B | B | B | A | A | B |
| 10091656068 | 2018-06-30 16:42:07 | 2018-06-30 16:48:14 | B | B | B | A | A | B | A | A | B | A | A | B | B | A | B | B |
| 10088679320 | 2018-06-28 17:42:37 | 2018-06-28 17:54:27 | B | B | B | B | A | A | A | A | B | A | A | B | B | A | B | B |
| 10087937746 | 2018-06-28 9:59:09 | 2018-06-28 10:12:06 | A | A | B | B | A | B | A | A | B | A | B | A | B | A | A | A |
| 10087914603 | 2018-06-28 9:38:05 | 2018-06-28 9:45:35 | A | B | B | B | A | B | A | B | A | A | B | A | A | A | A | A |
| 10087906836 | 2018-06-28 9:31:29 | 2018-06-28 9:38:38 | A | B | B | B | B | B | A | A | A | A | B | A | B | A | B | B |
| 10084210992 | 2018-06-26 12:18:00 | 2018-06-26 12:22:23 | A | A | B | B | A | B | A | A | B | A | A | B | B | A | B | B |
| 10082815414 | 2018-06-25 16:50:40 | 2018-06-25 17:00:43 | B | B | B | A | A | B | A | A | B | A | B | B | B | A | A | B |
| 10079689541 | 2018-06-22 17:22:18 | 2018-06-22 17:33:02 | B | A | A | B | A | B | A | B | A | A | B | A | B | A | A | B |
| 10077059309 | 2018-06-21 8:14:58 | 2018-06-21 8:27:56 | B | A | B | B | A | B | A | A | A | A | B | B | B | A | B | B |
| 10076325877 | 2018-06-20 21:09:09 | 2018-06-20 21:15:41 | A | A | B | B | A | B | A | B | A | A | B | A | A | A | A | A |
| 10074093192 | 2018-06-19 19:04:42 | 2018-06-19 19:06:34 | A | A | B | A | A | B | A | B | A | A | B | A | A | A | A | A |
| 10072386533 | 2018-06-18 21:23:08 | 2018-06-18 21:26:02 | A | B | A | B | B | A | A | A | A | A | B | B | B | A | B | B |
| 10070627809 | 2018-06-17 18:12:02 | 2018-06-17 18:25:56 | B | B | B | B | A | B | A | A | B | A | B | B | B | A | B | B |
| 10068090529 | 2018-06-15 9:21:02 | 2018-06-15 9:27:17 | B | B | B | B | B | B | B | B | B | B | B | A | A | B | B | B |
| 10067945674 | 2018-06-15 6:26:37 | 2018-06-15 6:37:25 | B | B | B | B | A | B | A | A | A | A | B | A | B | A | A | A |
| 10067797142 | 2018-06-15 3:27:31 | 2018-06-15 3:36:16 | B | B | A | A | A | B | A | A | B | A | B | B | B | A | B | B |
| 10067465139 | 2018-06-14 22:28:24 | 2018-06-14 22:33:44 | B | B | A | B | B | A | B | A | B | B | B | B | B | A | B | B |
| 10067342514 | 2018-06-14 21:09:54 | 2018-06-14 21:18:23 | B | B | B | B | A | A | A | A | B | A | A | A | B | A | A | B |
| 10066320470 | 2018-06-14 12:06:22 | 2018-06-14 12:16:15 | B | A | A | B | B | A | A | A | A | A | B | B | B | A | B | B |
